# Cost-effectiveness of palbociclib plus fulvestrant as second-line therapy of women with HR+/HER2- advanced breast cancer - A Chinese healthcare system perspective

**DOI:** 10.3389/fonc.2023.1068463

**Published:** 2023-03-14

**Authors:** Wentao Zhu, Miaomiao Zheng, Panpan Xia, Wanglong Hong, Guoqiang Ma, Aizong Shen

**Affiliations:** ^1^ School of Pharmacy, Anhui University of Chinese Medicine, Hefei, China; ^2^ Department of Pharmacy, the First Affiliated Hospital of University of Science and Technology of China (Anhui Provincial Hospital), Hefei, China

**Keywords:** cost-effectiveness, palbociclib, fulvestrant, advanced breast cancer, Markov model

## Abstract

**Aim:**

To evaluate the cost-effectiveness of palbociclib plus fulvestrant in the second-line treatment of women with hormone receptor-positive and human epidermal growth factor receptor 2-negative advanced breast cancer based on the latest published follow-up data from the perspective of the Chinese healthcare system.

**Methods:**

In view of the PALOMA-3 trial, a Markov model was built for this purpose, which included three health states: progression-free survival (PFS), progressed disease (PD), and death. The cost and health utilities were mainly derived from the published literature. One-way sensitivity analysis and probabilistic sensitivity analysis were carried out to verify the robustness of the model.

**Results:**

In the base case analysis, compared with the placebo plus fulvestrant arm, the palbociclib plus fulvestrant arm yielded an additional 0.65 quality-adjusted life years (QALYs) (2.56 QALYs vs. 1.90 QALYs) with an incremental cost of $36,139.94 ($55,482.06 vs. $19,342.12), resulting an incremental cost-effectiveness ratio (ICER) of $55,224.90/QALY, which was deeply higher than a willingness-to-pay (WTP) threshold of $34,138.28 per QALY in China. The results of one-way sensitivity analysis indicated that the utility of PFS, cost of palbociclib, and cost of neutropenia had a great influence on the ICER.

**Conclusions:**

Palbociclib plus fulvestrant is unlikely to be cost-effective in comparison with placebo plus fulvestrant as second-line therapy of women with HR+/HER2- advanced breast cancer.

## Introduction

Breast cancer is a type of cancer that is highly prevalent among female cancer patients. The latest global cancer burden data for 2020 released by the World Health Organization’s International Agency for Research on Cancer (IARC) shows that there are as many as 2.26 million new cases of breast cancer worldwide (1), accounting for 11.7% of all new cancer cases, which has surpassed lung cancer as the world’s number one cancer. In 2020, the number of new cases in China reaches 420,000, and the number of new breast cancer patients is expected to increase year by year in the future (2). Among them, hormone receptor-positive (HR+)/human epidermal growth factor receptor 2-negative (HER2-) patients were the most numerous. With medical advances, there have been significant improvements in the way cancer is treated. Although endocrine therapy has been tested and proven to be effective in patients with progressive advanced breast cancer, the ensuing drug resistance has become a major problem. The creation of cyclin-dependent kinases 4/6 (CDK4/6) inhibitors has brought hope for treatment. Clinical trials of CDK4/6 inhibitors have shown that endocrine combination targeted therapy has better prognostic value and effectively delays drug resistance (3).

Palbociclib is the first marketed cell CDK4/6 inhibitor that exerts its antitumor effects by inhibiting the binding of CDK4/6 and cell cycle protein D, interfering with retinoblastoma protein (RB) phosphorylation, and reducing cell proliferation in breast cancer cell lines (4). Fulvestrant is a selective estrogen receptor down-regulator endocrine drug that can completely inhibit estrogen receptor signaling and downstream signaling pathways, preventing estrogen from binding to tumor cells (5). Fulvestrant plus CDK4/6 inhibitors are recommended as the preferred second- and subsequent-line treatment for HR+/HER2- advanced breast cancer, subject to the most updated National Comprehensive Cancer Network (NCCN) guideline 2020 (6). Recently, a clinical trial (PALOMA-3) indicated that compared with placebo plus fulvestrant, palbociclib plus fulvestrant significantly extended progression-free survival [11.2 vs. 4.6 months; hazard ratio (HR) 0.50; 95% Cl,0.40-0.62; *P*<0.0001] (7). Because there was no significant difference in overall survival, the researchers prolonged the follow-up of the trial, with the latest data showing that after a median follow-up of 73.3 months, the median overall survival was 34.8 months and 28.0 months in the palbociclib group and placebo group [stratified risk ratio 0.81; 95% Cl,0.65-0.99; *P*=0.0122]. The six-year overall survival rates were 19.1% and 12.9% in the palbociclib and placebo groups, respectively (8). Thus, palbociclib in combination with fulvestrant was shown to have a conspicuous median overall survival benefit with no obvious difference and could be an option for second-line treatment of HR+/HER2- advanced breast cancer (9).

Although the PALOMA-3 trial has demonstrated the excellent safety and efficacy of palbociclib plus fulvestrant, there are no studies of the economic value of this treatment option with the latest follow-up data (8). Therefore, the purpose of our analysis was to explore the cost-effectiveness of palbociclib plus fulvestrant in the second-line treatment of women with HR+/HER2- advanced breast cancer based on a dynamic Markov model from the perspective of the Chinese healthcare system.

## Methods

### Analytical overview

The target population enrollment criteria for this study were derived from the PALOMA-3 trial (NCT01942135), an international randomized, double-blind, placebo-controlled trial. A total of 521 patients were randomized 2∶1 to receive palbociclib plus fulvestrant or placebo plus fulvestrant, of which palbociclib at a dose of 125 mg orally for 3 weeks once daily, followed by 1 week off for a total cycle of 28 days and fulvestrant at a dose of 500 mg intramuscularly, every 2 weeks for the first three injections followed by every 4 weeks (7).

### Model structure

**Figure 1:**
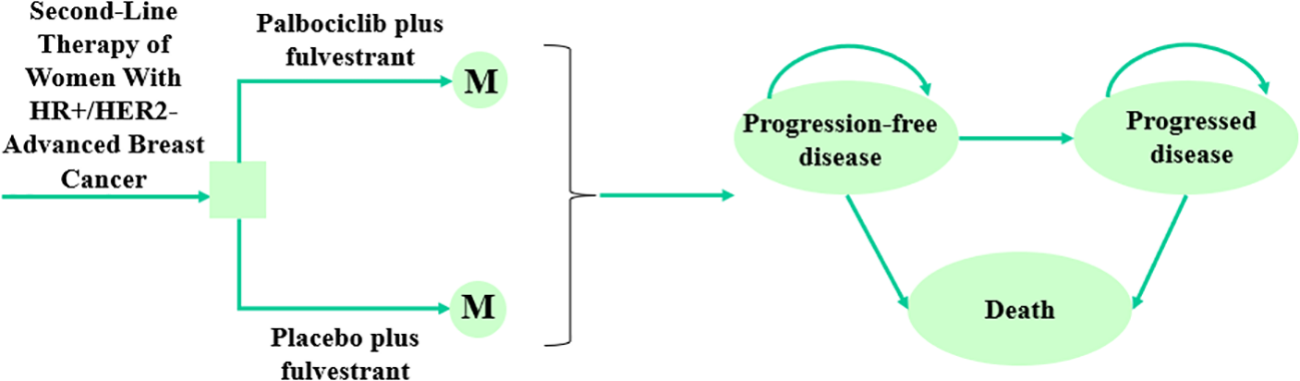
The Markov model structure and the decision tree of palbociclib plus fulvestrant versus placebo plus fulvestrant as second-line therapy of women with HR+/HER2- advanced breast cancer. HR+, Hormone receptor-positive; HER2-, Human epidermal growth factor receptor 2-negative.


In this study, a Markov model was constructed to analyze health and economic outcomes (10), which included three exclusive health states: progression-free survival (PFS), progressed disease (PD), and death (**Figure 1**). It was assumed that all patients were in the PFS state at entry into the model and entered the progression or death state depending on the probability of metastasis, or stayed in the PFS state. Ultimately, death was an absorption state. In addition, we performed a half-cycle correction to reduce the error. Model transfer probabilities were obtained by Survival analysis. After 10 years, 99% of the patients were dead, so we chose 10 years as a suitable time horizon. And the cycle length of the Markov model was set to 28 days, which corresponded to the treatment cycle length. The main model outcomes were the total cost, quality-adjusted life years (QALYs), and incremental cost-effectiveness ratios (ICERs). And the discount rate for both cost and utility was 5%. The formula for calculating ICER was as follows (11):

$$\text{ICER=}\frac{\text{[cost}\left(\text{palbociclib plus fulvestrant arm}\right)\text{-cost(placebo plus fulvestrant arm)]}}{\text{[effectiveness(palbociclib plus fulvestrant arm)-effectiveness(placebo plus fulvestrant arm)]}}$$

### Clinical data

The estimates of PFS and OS were obtained from the newly published PALOMA-3 trial (8). Firstly, the GetData Graph Digitizer software (version 2.26) was used to extract the survival data of patients from the Kaplan-Meier (KM) survival curves. Secondly, these data points were applied to fit several different parametric survival functions including Weibull, exponential, log-normal, log-logistic, Gompertz, and generalized gamma. On the basis of the Akaike Information Criterion (AIC) and Bayesian Information Criterion (BIC), Weibull distributions were determined to be the most reasonable functions to extrapolate the PFS and OS (12). Finally, the scale parameter (λ) and shape parameter (γ) were obtained using R software (version 4.1.0), detailed data were demonstrated in **Table 1**. Survival function in terms of Weibull distribution at t was *S*(*t*)=exp(-*λt* ^γ^) (t represents the number of cycle in the Markov model). And then the transition probability was calculated based on the following formula: P(*t*)=1-exp[*λ*(*t*-1)^γ^- λ*t* ^γ^]. Moreover, we assumed that the transition probability from the PFS state to the Death state is the natural mortality rate of the Chinese population in 2021 (7.18‰) (17).

**Table 1:** Model parameters.

Parameter	Base case	Lower	Upper	Distribution	References
Weibull survival model input					
PFS in Palbociclib plus fulvestrant arm	Shape=0.95501 Scale=0.06497	–	–	–	(7, 8)
OS in Palbociclib plus fulvestrant arm	Shape=1.30497 Scale=0.00656	–	–	–	(7, 8)
PFS in Placebo plus fulvestrant arm	Shape=0.8904 Scale=0.1405	–	–	–	(7, 8)
OS in Placebo plus fulvestrant arm	Shape=1.34619 Scale=0.00727	–	–	–	(7, 8)
Direct cost, $					
Palbociclib	640.39/125mg	525.12	768.49	Gamma	Local charge
Fulvestrant	287.90/250mg	233.77	338.71	Gamma	Local charge
Follow-up	166/cycle	132.8	193.02	Gamma	(13)
Drug administration	33.56/cycle	28.27	40.27	Gamma	(14)
Supportive care	807/cycle	690.39	953.34	Gamma	(13)
Terminal care	1893/once	1533.33	2209.13	Gamma	(13)
Cost of AEs, $					
Neutropenia	412	333.72	468.18	Gamma	(13)
Leukopenia	435.58	378.95	537.75	Gamma	(15)
Infections	395.82	351.49	494.77	Gamma	(15)
Fatigue	110	89.50	129.41	Gamma	(13)
Nausea	323	261.63	375.58	Gamma	(13)
Incidence of palbociclib plus fulvestrant arm AEs (grade≥3)					
Neutropenia	0.577	0.467	0.704	Beta	(7, 8)
Leukopenia	0.377	0.318	0.459	Beta	(7, 8)
Infections	0.046	0.041	0.055	Beta	(7, 8)
Fatigue	0.029	0.024	0.034	Beta	(7, 8)
Nausea	0.006	0.005	0.007	Beta	(7, 8)
Incidence of placebo plus fulvestrant arm AEs (grade≥3)					
Leukopenia	0.006	0.005	0.007	Beta	(7, 8)
Infections	0.035	0.028	0.043	Beta	(7, 8)
Fatigue	0.012	0.01	0.015	Beta	(7, 8)
Nausea	0.006	0.005	0.007	Beta	(7, 8)
Utility inputs					
Utility of PFS	0.87	0.72	0.96	Beta	(16)
Utility of PD	0.71	0.58	0.82	Beta	(16)
Discount rate	0.05	0	0.08	Normal	

PFS, Progression-free survival; OS, Overall survival; PD, Progressed disease.

### Cost and utility inputs

This study was conducted from the perspective of the Chinese healthcare system, only direct medical costs such as drug acquisition costs, follow-up costs, drug administration costs, supportive care costs, terminal care costs, and AEs management costs were considered (**Table 1**). In addition, all costs were calculated in United States dollars at an exchange rate of 7.116 RMB per the United States dollars (October 2022) (18) and were adjusted, if necessary, based on the medical care consumer price index (CPI). Drug acquisition costs were from the median price of the bid-winning drug price from Menet (https://www.menet.com.cn/), China’s leading platform for pharmaceutical and health information. Other costs for medical services were obtained from previous literature. To demonstrate the credibility of the costs, they were compared with the prices of medical services published by Chinese government agencies, and no significant differences were found. Moreover, we only considered adverse events (grade≥3), including neutropenia, leukopenia, infections, fatigue, and nausea. The incidence rates of AEs came from the PALOMA-3 trial (7, 8).

Since utility values were not reported in the PALOMA-3 trial, the utility value data used in this paper are from the previously published study (16), which used the EuroQoL 5-dimension 5-level (EQ-5D-5L) scale to measure the health utility values of Chinese breast cancer patients, and the utility values for PFS and PD were 0.87 and 0.71, respectively.

### Willingness-to-pay threshold

There is no unified standard for willingness-to-pay (WTP) thresholds worldwide, but WTP thresholds are evaluation criteria for judging whether a treatment option is economic, and World Health Organization (WHO) recommends using per capita gross domestic product (GDP) as the threshold (19), considering the uneven economic level of each country. Hence, this study refers to the recommendations of WHO and China Guidelines for Pharmacoeconomic Evaluation 2020 (20), three times the GDP per capita of China in 2021 ($34,138.28) is set as the WTP threshold (21).

### Sensitivity analysis

The robustness of the model parameters and assumptions was verified by deterministic sensitivity analysis (DSA) and probabilistic sensitivity analysis (PSA) so that the uncertainty in the model could be justified to the maximum extent. In the one-way sensitivity analysis, we kept the other parameters fixed at the baseline values and assumed that a single parameter varies within a certain range, usually taking 20% of the baseline value as the upper and lower limits. For the sake of considering uncertainty, the effect of parameter changes on the ICER was calculated. For the probabilistic sensitivity analysis, we performed 1000 Monte Carlo simulations to evaluate the parameter uncertainty by setting a specific probability distribution for each parameter. Gamma distribution was used for costs and Beta distribution was used for utilities and probabilities (22). The results of 1000 Monte Carlo simulations were presented as cost-effectiveness scatter plots and cost-effectiveness acceptability curves.

## Results

### Base case results

The results of the base case were shown in **Table 2**, where the palbociclib plus fulvestrant arm had higher costs and utilities compared to the placebo plus fulvestrant arm after ten years of model simulation. In comparison with the placebo plus fulvestrant arm, the incremental cost of the palbociclib plus fulvestrant arm was $36,139.94 ($55,482.06 vs. $19,342.12) and the incremental utility was 0.65 QALYs (2.56 QALYs vs. 1.90 QALYs), resulting in the ICER of $55,224.90/QALY, which indicated that palbociclib plus fulvestrant was not economical at the WTP threshold of $34,138.28 per QALY in China.

**Table 2:** Base case results.

Treatment	Costs ($)	Incremental Costs ($)	QALYs	Incremental QALYs	ICER($/QALY)
Palbociclib plus fulvestrant arm	55482.06	36139.94	2.56	0.65	55224.90
Placebo plus fulvestrant arm	19342.12		1.90		

QALY, Quality-adjusted life year; ICER, Incremental cost-effectiveness ratio.

### Sensitivity analysis

**Figure 2:**
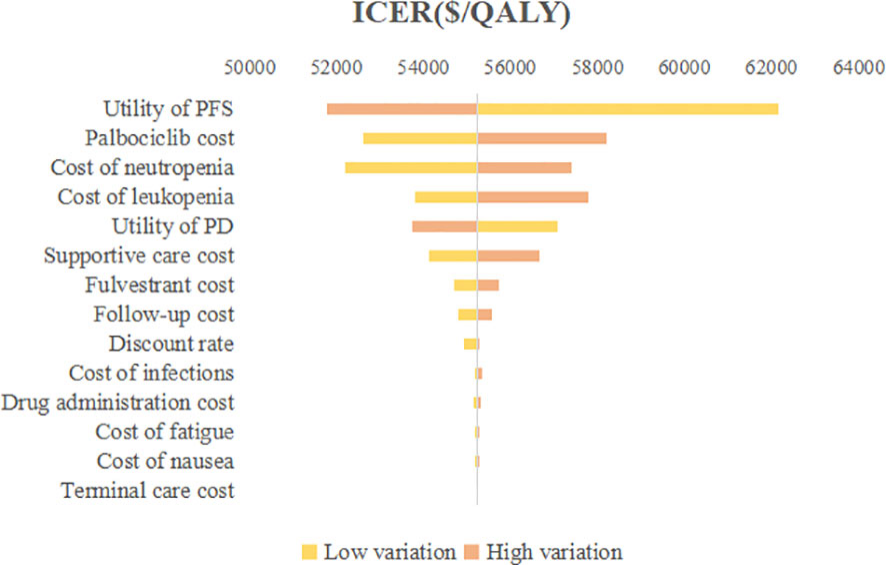
Tornado diagram of one-way sensitivity analysis. ICER, Incremental cost-effectiveness ratio; QALY, Quality-adjusted life year; PFS, Progression-free survival; PD, Progressed disease.

The outcomes of one-way sensitivity analysis shown in tornado diagram (**Figure 2**), revealed that the utility of PFS, cost of palbociclib, and cost of neutropenia had a great impact on the results of the ICER. On the contrary, the other parameters made a mild difference in the ICER. However, no matter how the parameters were changed, they did not cause the ICER to be lower than the WTP threshold ($34,138.28). Palbociclib prolonged the PFS of patients, improved quality of life, and had superior clinical outcomes, so the utility value of PFS played a vital role in outcomes. To summarize, none of these parameters substantially changed the economic results.

**Figure 3:**
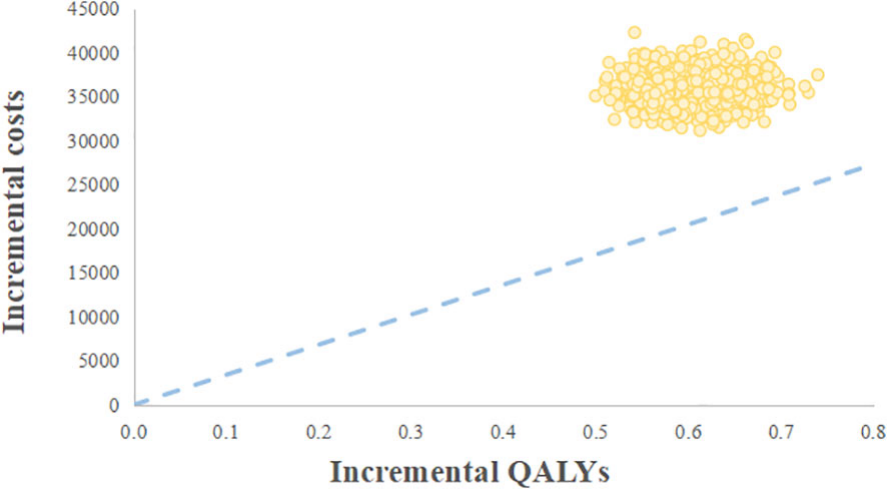
Cost-effectiveness scatter plot. WTP, Willingness-to-pay; QALY, Quality-adjusted life year.

**Figure 4:**
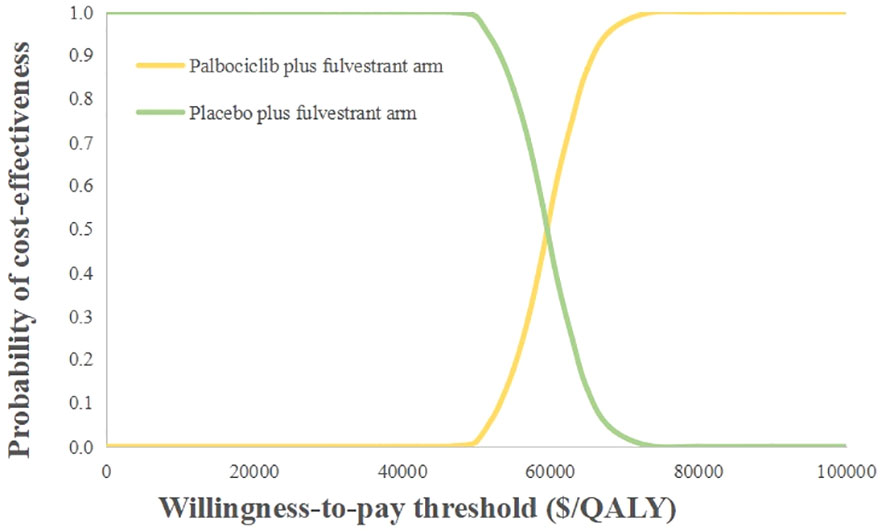
Cost-effectiveness acceptability curves. QALY, Quality-adjusted life year.

The probabilistic sensitivity analysis reflected that the probability of the palbociclib plus fulvestrant arm being cost-effective was 0 when using 3 times the GDP per capita of China in 2021 ($34,138.28) as the willingness-to-pay threshold (**Figure 3**). Cost-effectiveness acceptability curves (**Figure 4**) showed that the probability of having a cost-effective advantage increased with growing WTP thresholds. When the WTP thresholds were $59,581.59/QALY and $78,048.90/QALY, in contrast to placebo plus fulvestrant, the probabilities of the cost-effectiveness of palbociclib plus fulvestrant were 50% and 100%, respectively.

## Discussion

HR+/HER2- advanced breast cancer accounts for approximately 70% of all breast cancer patients, and disease progression in this group is dependent on sex hormone regulation, making endocrine therapy the preferred option (23). If the disease progresses after endocrine therapy, the drug used for endocrine therapy can usually be changed or endocrine therapy combined with targeted therapy. The successive emergence of palbociclib, ribociclib, and abemaciclib has shown good therapeutic effects of CDK4/6 inhibitors (24, 25). Consequently, it is necessary for patients to choose treatment options that are more effective and less costly in accordance with their conditions (26).

To our knowledge, this current study is the first cost-effectiveness analysis of palbociclib plus fulvestrant for the second-line treatment of HR+/HER2- advanced breast cancer from the Chinese healthcare system based on the newly published data. According to our base case analysis, the palbociclib plus fulvestrant provided 2.56 QALYs, with a total cost of $55482.06. The placebo plus fulvestrant provided 1.90 QALYs at a total cost of $19342.12. Causing an ICERs of $55,224.90 per QALY, which extremely exceeds a WTP threshold of $34,138.28 per QALY in China. The results of sensitivity analysis found that the utility value of PFS had the greatest effect on ICER, then the cost of palbociclib, neutropenia treatment cost, and leukopenia treatment cost also had greater impacts on findings. Nevertheless, the analysis was relatively good in terms of robustness. Probabilistic sensitivity analysis suggested that the palbociclib plus fulvestrant arm had zero chance to be cost-effective in China.

China’s antineoplastic drug market has been on a steady growth trend in recent years. From the outcomes of medical insurance negotiations announced by National Healthcare Security Administration in 2021, the average price reduction of drugs outside the medical insurance catalog was 61.71%, which bagged a national record haul (27). Among them, antineoplastic drugs became the most insured drugs. Eribulin mesylate and neratinib for the treatment of breast cancer also entered the medical insurance catalog. The average price reduction for these antineoplastic drugs was 64.88%, exceeding the overall level. Although China has launched a series of measures such as centralized drug procurement and national drug price negotiation, the accessibility and affordability of antineoplastic drugs for patients have been improved, but there were still many drugs that were expensive and patients faced greater financial pressure.

In recent years, the medical level has advanced and developed rapidly, and we have made great breakthroughs in cancer treatment modalities, such as targeted drugs and immunotherapy, which have effectively improved the quality of patients’ survival, but the expensive medical costs have also imposed a heavy financial burden on many patients. Besides, in addition to the antineoplastic drugs being added to China’s national medical insurance catalog by national medical insurance system, in order to improve the affordability of drugs and to reduce the burden on patients, the Patient Assistance Program (PAP) is often launched in China after new drugs come into the market. PAP is a program where low-income or poor cancer patients who meet the medical or financial criteria of the program can apply to receive free medication and free medication after purchase (28). For example, in an assistance program implemented by the Cancer Foundation of China in 2021, patients who measure up the criteria can pay for up to six out-of-pocket payments of Nivolumab in the application year and receive assistance for all remaining drugs in that application year, reducing the annual cost of treatment to ¥110,000, the drop was more than 75%. It is clear that PAP is an initiative that benefits the majority of patients and effectively alleviates their poverty due to cancer. As can be seen from the sample given, there would be an excellent cost-effectiveness advantage if the palbociclib was covered by PAP.

In this study, we used three times the GDP per capita of China in 2021 ($34,138.28) as the cost-effectiveness threshold, considering that China’s regional development level was uneven and there were large differences in GDPs. For example, Beijing’s triple the GDP per capita in 2021 was $77,529.51, Shanghai’s triple the GDP per capita was $73,271.50, and Jiangsu’s triple the GDP per capita was $57,883.64 (21). It was perfectly cost-effective to use three times the GDP per capita of these three provinces and cities as the threshold. As a consequence, the choice of the threshold was crucial, and it directly affected the last economy.

Admittedly, there were several limitations in this analysis. First, this study was performed by fitting parameter distributions to obtain PFS and OS data of patients, which led to uncertainty in the results and reflected on the robustness of the model despite the fact that the parameters have been validated. Second, the utility value data in this analysis were derived from a study of Chinese breast cancer patients with health utility value measures, which included patients with similar sociodemographic and clinical characteristics as in this paper. However, there was no standard utility score system for the EQ-5D-5L scale in China, and this study used the British utility score conversion system (29). Since there were differences in culture, race, attitude, and preference in each country, which may affect the ultimate utility value. Although the outcomes of the one-way sensitivity analysis showed that the utility value of PFS made the greatest difference in the ICER, we could see from the tornado diagram that changes in the PFS did not cause the final results to flip. Third, the drug costs in this study were derived from the median drug prices in Jiangsu Province, China, which varied slightly by province. Most of the other parameters were from published literature and not real-world data. Clinical practice tended to be more diverse, and the parameters we used were a summary of general patterns for most patients. The actual situation needed to be studied in depth with the real-world data. Fourth, we only included grade three or four adverse events and ignored the adverse events below grade three, which might underestimate the total cost of the treatment (30).

Our analysis is informative for palbociclib to enter the medical insurance catalog or be covered by the PAP, and we hope to conduct more real-world clinical studies in the future so that our economic evaluation will be more accurate.

## Conclusions

As described, although palbociclib plus fulvestrant showed wonderful clinical efficacy as second-line therapy of women with HR+/HER2- advanced breast cancer, these estimates suggested that palbociclib plus fulvestrant arm, compared with placebo plus fulvestrant arm, is unlikely to be cost-effective from the perspective of the Chinese healthcare system at the WTP threshold of $34,138.28 per QALY in China.

## Data availability statement

The original contributions presented in the study are included in the article/supplementary files, further inquiries can be directed to the corresponding author/s.

## Author contributions

WZ and AS conceived the experiments. WZ, MZ, PX, and AS developed the economic model and performed the analyses. WZ, WH, and GM collected and reviewed the data. WZ and MZ wrote the first draft of the manuscript. PX, WH, and GM performed first draft refinement. All authors contributed to the article and approved the submitted version.
